# Analysis of the Optimum Gain of a High-Pass L-Matching Network for Rectennas

**DOI:** 10.3390/s17081712

**Published:** 2017-07-25

**Authors:** Manel Gasulla, Josep Jordana, Francesc-Josep Robert, Jordi Berenguer

**Affiliations:** 1 e-CAT Reaserch Group, Department of Electronic Engineering, Castelldefels School of Telecommunications and Aerospace Engineering, Universitat Politècnica de Catalunya, c/Esteve Terradas, 7, 08860 Castelldefels (Barcelona), Spain; jose.jordana@upc.edu (J.J.); francesc.j.robert@upc.edu (F.-J.R.); 2 CSC Research Group, Department of Signal Theory and Communications, Castelldefels School of Telecommunications and Aerospace Engineering, Universitat Politècnica de Catalunya, c/Esteve Terradas, 7, 08860 Castelldefels (Barcelona), Spain; jordi.berenguer@upc.edu

**Keywords:** RF harvesting, rectenna, low-power management, L-matching network, optimum voltage gain, internet of things

## Abstract

Rectennas, which mainly consist of an antenna, matching network, and rectifier, are used to harvest radiofrequency energy in order to power tiny sensor nodes, e.g., the nodes of the Internet of Things. This paper demonstrates for the first time, the existence of an optimum voltage gain for high-pass L-matching networks used in rectennas by deriving an analytical expression. The optimum gain is that which leads to maximum power efficiency of the rectenna. Here, apart from the L-matching network, a Schottky single-diode rectifier was used for the rectenna, which was optimized at 868 MHz for a power range from −30 dBm to −10 dBm. As the theoretical expression depends on parameters not very well-known a priori, an accurate search of the optimum gain for each power level was performed via simulations. Experimental results show remarkable power efficiencies ranging from 16% at −30 dBm to 55% at −10 dBm, which are for almost all the tested power levels the highest published in the literature for similar designs.

## 1. Introduction

Radio frequency (RF) energy harvesting has been widely proposed to power tiny devices such as RFID tags, autonomous sensors, or IoT (Internet of Things) nodes [1,2,3,4,5,6,7,8,9]. RF energy can be harvested either from dedicated sources, such as in the case of RFID devices, or from the RF energy already present in the ambient and coming from unintentional sources such as TV, FM radio, cellular, or WiFi emitters.

**Figure 1:**
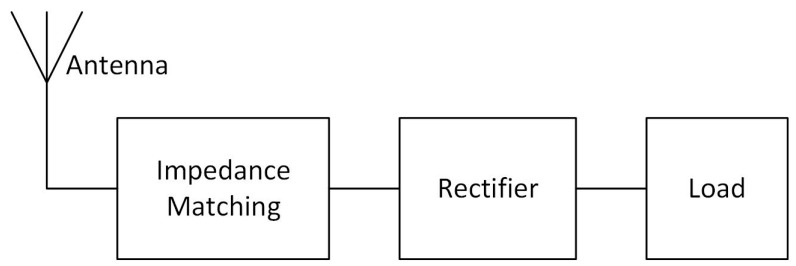
Block diagram of a rectenna with an output load.

In order to harvest RF energy, a rectenna (rectifying antenna) is used. Figure 1 shows the block diagram of a conventional rectenna, consisting of an antenna, an impedance matching network and a rectifier. The rectifier provides a suitable DC voltage in order to power a load, e.g., an RFID tag or IoT node, and the matching network matches the output impedance of the antenna to the equivalent impedance at the input of the rectifier in order to transfer the available maximum power.

As the available power at the antenna decreases so does the generated voltage. Whenever this voltage is not high enough to properly bias the diodes of the rectifier, the power efficiency decreases severely. Several techniques have been proposed in order to increase the efficiency at low power levels. For example, in [1,2,10] dual-stage solutions were proposed, where each of the two implemented circuits was optimized for different ranges of input power. In [11], the size of the diode-connected MOS transistors was optimized. On the other hand, in order to reduce the voltage drop of the diodes, novel devices have been proposed such as MOS floating-gate devices [12], tunnel FETs [13], or MOS transistors with a new bulk connection [14]. Another widely used technique consists in using the matching network for boosting the voltage at the rectifier input [4,8,14,15,16,17,18,19,20,21,22,23,24,25,26]. Among them, one of the simplest and most widely used is the L-matching network, where the voltage gain is fixed by the resistance value of the load.

This paper demonstrates for the first time the existence of an optimum voltage gain for a high-pass L-matching network used in rectennas by deriving an analytical expression. The optimum gain is that which leads to maximum power efficiency of the rectenna. As the focus is on the matching network, a rectifier with a single series diode configuration was selected for simplicity. Input power levels in the range of −30 dBm to −10 dBm at the 868 MHz Short Range Devices (SRD) band were considered. At each power level, the value provided by the expression of the optimum gain was used as the initial point for an ensuing accurate search via simulations. The rectenna was later implemented and experimental results show remarkable power efficiencies compared with other works with similar designs found in the literature.

The paper is organized as follows: Section 2 presents a theoretical analysis of the rectenna where the analytical expression of the optimum voltage gain of the matching network is derived. Section 3 shows simulation results of the rectenna with the Keysight ADS software for obtaining the optimum gain and components of the matching network that will be used in the implementation of the circuit. In Section 4 the performance of the implemented rectenna is presented. Section 5 concludes the work and two appendices present supplemental material.

## 2. Theoretical Analysis of the Rectenna

This section presents the analysis of the rectenna with both an ideal (Section 2.1) and a lossy (Section 2.2) matching network. The optimum voltage gain of the matching network is derived in Section 2.2.

### 2.1. Rectenna with Ideal Matching Network

**Figure 2:**
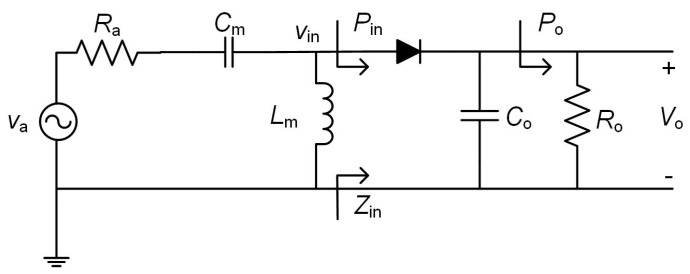
Proposed rectenna.

Figure 2 shows the circuit schematic of the proposed rectenna, which includes a high pass L-matching network (composed of a capacitor *C*_m_ and an inductor *L*_m_), a half-wave rectifier and an output filtering capacitor (*C*_o_) and load (*R*_o_). The antenna is modelled by a sinusoidal voltage source *v*_a_ with a series radiation resistance *R*_a_. On the other hand, *v*_in_, *Z*_in_ and *P*_in_ respectively are the voltage, impedance and power at the input of the rectifier, and *V*_o_ and *P*_o_ respectively are the DC voltage and power at the load.

The voltage amplitude (or peak voltage) of *v*_a_ is given by [17]:

(1) $$V_{\mathrm{ap}}=2\sqrt{2R_{a}P_{\mathrm{av}}},$$

where *P*_av_ is the available power at the antenna. On the other hand, the overall power efficiency of the rectenna is defined as:

(2) $$\eta_{\mathrm{rect}}=\eta_{\mathrm{in}}\eta_{o},$$

where the input efficiency is given by:

(3) $$\eta_{\mathrm{in}}=\frac{P_{\mathrm{in}}}{P_{\mathrm{av}}},$$

and the rectifier efficiency is:

(4) $$\eta_{o}=\frac{P_{o}}{P_{\mathrm{in}}}=1-\frac{V_{\gamma }}{V_{\mathrm{inp}}},$$

being *V*_inp_ the amplitude voltage of *v*_in_ and *V*_γ_ the threshold forward voltage of the diode (assumed constant here). Equation (4) is a simplistic approximation that neglects all the parasitic components and non idealities of the diode except *V*_γ_.

**Figure 3:**
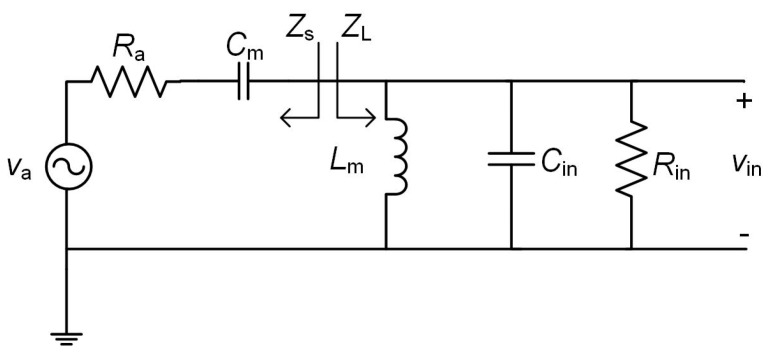
Equivalent circuit of the rectenna.

In order to achieve a high value of *η*_in_ (ideally 1), *Z*_in_ has to be matched to *R*_a_. On the other hand, from Equation (4), lower values of *V*_γ_ and higher values of *V*_inp_ lead to a higher value of *η*_o_. Thus, *η*_o_ can be increased, for example, by using Schottky diodes (low *V*_γ_) and a matching network with a high voltage gain (high *V*_inp_). In order to illustrate how the matching network provides this gain, the circuit of Figure 2 is transformed into the circuit of Figure 3, where *C*_in_ and *R*_in_ model *Z*_in_ in Figure 2.

*C*_in_ is mainly due to the parasitic capacitance of the diode whenever *C*_o_ is much larger, which is usually the case, and *R*_in_, which models the power delivered to the rectifier input, is given by [27]:

(5) $$R_{\mathrm{in}}=\frac{R_{o}}{2}\frac{1}{1-V_{\gamma }/V_{\mathrm{inp}}}\;.$$

In order to transfer the maximum power to the rectifier input (*P*_in_ = *P*_av_ and thus *η*_in_ = 1), it must be accomplished that $Z_{L}=Z_{s}^{*}$, resulting in the following voltage gain of the matching network:

(6) $$G_{t}=\frac{V_{\mathrm{inp}}}{V_{\mathrm{ap}}}=\frac{1}{2}\sqrt{\frac{R_{\mathrm{in}}}{R_{a}}}=\frac{1}{2}\sqrt{\left(1+Q^{2}\right)},$$

where *Q* is the quality factor of the circuit. Appendix A shows the resulting expressions for *C*_m_ and *L*_m_ as well graphs of these parameters in function of *G*_t_.

As can be seen from Equation (6), *G*_t_ depends on the relationship between *R*_in_ and *R*_a_, so the gain *G*_t_ can be made arbitrarily large by increasing *R*_in_. Expression (6) can also be derived equating the power at the input of the matching network with that dissipated in *R*_in_, assuming a lossless matching network [16]. Thus, an increase of *R*_in_ requires a square increase of *v*_in_ (and thus of *G*_t_) to keep power constant. On the other hand, *R*_in_ can be increased, from Equation (5), by increasing *R*_o_. However, *R*_o_ is a priori fixed by the load to be powered, e.g., an IoT node. Fortunately, *R*_o_ can be arbitrarily and automatically changed by placing an additional impedance matching stage between the rectenna output and the load. Such stage, which is out of the scope of this work, is normally implemented by a maximum power point tracker, which has been extensively used in solar, thermal, and mechanical energy harvesters, but also in RF harvesters, such as in [28,29,30].

### 2.2. Rectenna with Lossy Matching Network: Optimum Voltage Gain

In the previous analysis, the losses of the matching network components have not been considered and thus will be taken into account next. As will be shown, their inclusion is significant and leads to the concept of optimum voltage gain of the matching network.

**Figure 4:**
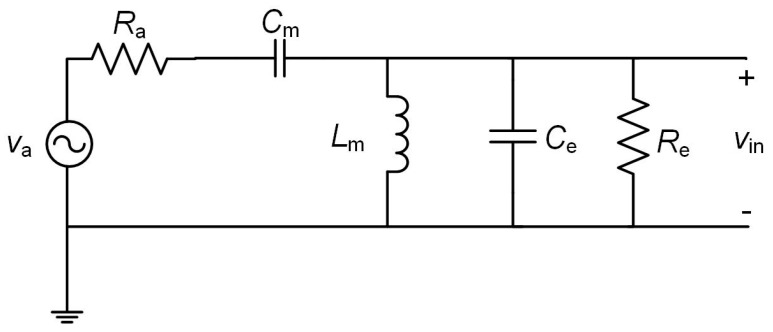
Equivalent circuit of the rectenna taking into account the inductor model of Figure A4 with *R*_2_ neglected.

In general, the parasitic loss of capacitors is very small compared with that of inductors [16,31,32] and will be neglected in the analysis. Then, taking into account the inductor model of Appendix B, Figure 3 is transformed into Figure 4, where *L*_m_ ≈ $L_{m}^{\prime }$ and *C*_in_ and *R*_in_ have been substituted by *C*_e_ and *R*_e_. The resistance *R*_e_ includes the parasitic losses (*R*_p_) of the coil and is given by:

(7) $$\frac{1}{R_{e}}=\frac{1}{R_{p}}+\frac{1}{R_{\mathrm{in}}},$$

being:

(8) $$\frac{1}{R_{p}}=\frac{1}{R_{v}^{\prime }}+\frac{1}{R_{1}^{\prime }},$$

and *C*_e_ includes the parasitic capacitance of the coil and is given by:

(9) $$C_{e}=C_{\mathrm{in}}+C_{1}^{\prime },$$

For the circuit of Figure 4, the gain of the matching lossy network is:

(10) $$G_{t}=\frac{1}{2}\sqrt{\frac{R_{e}}{R_{a}}},$$

Now, even with a large value of *R*_in_, *R*_e_ and thus *G*_t_ will be limited by *R*_p_. Further, at matching conditions [16]:

(11) $$\eta_{\mathrm{in}}=\frac{R_{e}}{R_{\mathrm{in}}}=\frac{R_{p}}{R_{p}+R_{\mathrm{in}}},$$

as some power will be dissipated at *R*_p_. Therefore, large values of *R*_in_ (>>*R*_p_ and thus >>*R*_e_) decrease *η*_in_ without significantly increasing *G*_t_ and thus *η*_o_. Contrariwise, low values of *R*_in_ (≪*R*_p_ and thus *R*_in_ ≈ *R*_e_) decrease *G*_t_ and thus *η*_o_ without significantly increasing *η*_in_ (≈1). So, a trade-off exists for achieving a maximum value of *η*_rect_, which leads to an optimum *R*_in_ and *G*_t_.

The optimum value of *G*_t_ can be found by expressing Equation (2) in function of *G*_t_, equating its derivative to zero and finding the roots. First, operating from Equations (7) and (10), we obtain

(12) $$R_{\mathrm{in}}=\frac{4G_{t}^{2}R_{a}R_{p}}{R_{p}-4G_{t}^{2}R_{a}},$$

and substituting this in Equation (11) we get:

(13) $$\eta_{\mathrm{in}}=1-4G_{t}^{2}\frac{R_{a}}{R_{p}},$$

On the other hand, using Equation (6) in Equation (4) we arrive at:

(14) $$\eta_{o}=1-\frac{V_{\gamma }}{G_{t}V_{\mathrm{ap}}},$$

and thus from Equations (2), (13) and (14) we obtain:

(15) $$\eta_{\mathrm{rect}}=\left(1-4G_{t}^{2}\frac{R_{a}}{R_{p}}\right)\left(1-\frac{V_{\gamma }}{G_{t}V_{\mathrm{ap}}}\right),$$

**Figure 5:**
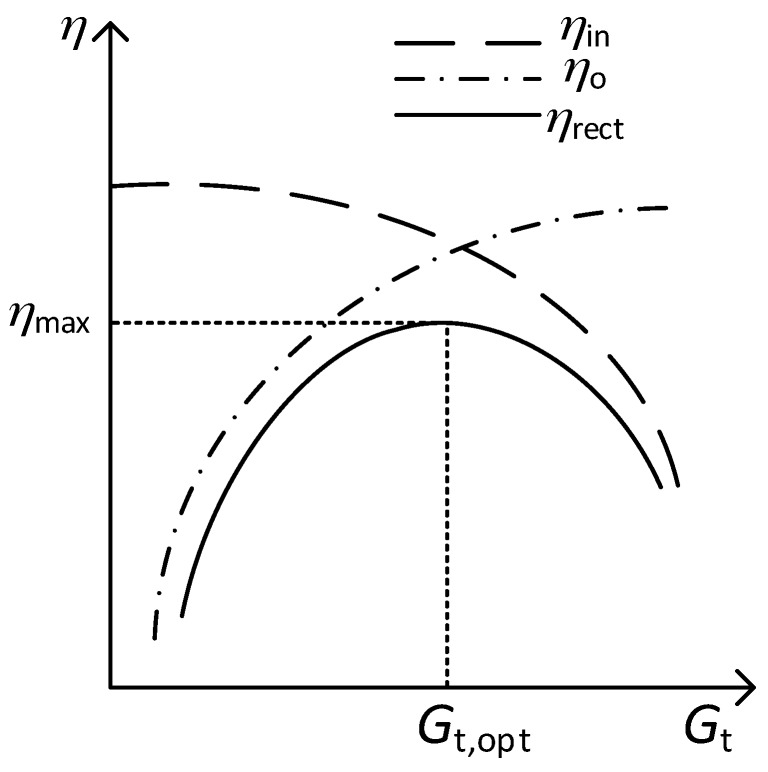
Qualitative graphs of the efficiencies of the rectenna versus *G*_t_.

Figure 5 shows a qualitative representation of Equations (13)–(15). As can be seen, *η*_in_ decreases and *η*_o_ increases with increasing values of *G*_t_, leading to an optimum value of *G*_t_ (*G*_t,opt_) that provides the maximum value of *η*_rect_ (*η*_max_).

The expression of *G*_t,opt_ can be reached by doing the derivative of Equation (15) with respect to *G*_t_ and equating to zero, thus arriving to the following third-degree equation:

(16) $$G_{t}^{3}-\frac{V_{\gamma }}{2V_{\mathrm{ap}}}G_{t}^{2}-\frac{R_{p}V_{\gamma }}{8R_{a}V_{\mathrm{ap}}}=0.$$

In this case, only a single positive real root results, which can be approximated to:

(17) $$G_{t,\mathrm{opt}}\approx \frac{1}{6}\frac{V_{\gamma }}{V_{\mathrm{ap}}}+\frac{1}{2}\sqrt[{3}]{\frac{V_{\gamma }}{V_{\mathrm{ap}}}\frac{R_{p}}{R_{a}}},$$

The derivation and expression for *G*_t,opt_ has not previously reported in the literature. As can be seen, *G*_t,opt_ increases with increasing values of *R*_p_ and *V*_γ_ and with decreasing values of *V*_ap_. This can be also inferred from the above expressions and Figure 5. Effectively, from Equation (13), an increase of *R*_p_ (decrease of inductor losses) increases *η*_in_, shifting upwards the corresponding curve in Figure 5 and thus to the right *G*_t,opt_ (higher value). At the same time, the value of *η*_max_ will increase. On the other hand, from Equation (14), a higher value of *V*_γ_ or a lower value of *V*_ap_ decrease *η*_o_, shifting downwards the corresponding curve in Figure 5 and thus again to the right *G*_t,opt_. In this case, though, *η*_max_ will decrease. So, *η*_max_ increases with increasing values of *R*_p_ and *V*_ap_ and with decreasing values of *V*_γ_.

In order to obtain Equation (17), the values of *V*_γ_ and *R*_p_ are required. However, *V*_γ_ depends on the current flowing through the diode, which depends on *P*_av_ but also on *G*_t_. On the other hand, *R*_p_ depends on the specific commercial component of *L*_m_, whose value, from Equation (A6), again depends on *G*_t_. Therefore, it is not straightforward obtaining *G*_t,opt_ and it will be found here by simulations, as shown in Section 3. Anyhow, the above derivation demonstrates the existence of an optimum gain value, provides more insight on the optimum gain and rectenna efficiency, and from Equation (17) an initial guess can be used for the simulations.

## 3. Rectenna Simulation Analysis 

**Figure 6:**
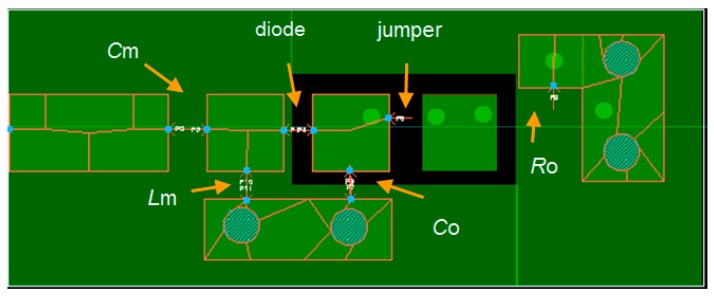
PCB Layout of the rectenna with indication to the placement of the components.

Simulations of the rectenna of Figure 2 have been carried out using the Keysight ADS software. The Harmonic Balance Analysis was used in order to compute the steady state solutions. For the diode, a Schottky HSMS-2850 device (Avago Technologies, Sant Jose, CA, USA) was selected, as it presents a low voltage drop (*V*_γ_ ≈ 0.1 V @ 0.1 mA) and a low capacitance (*C*_jo_ = 0.18 pF). Input power levels from −30 dBm to −10 dBm in steps of 5 dBm were used at a frequency of 868 MHz. Commercial components from the vendor libraries for *C*_m_ (AVX, Fountain Inn, SC, USA) and *L*_m_ (Coilcraft, Cary, IL, USA) were also used. A layout was also included in the simulations and the Momentum simulator was executed in order to obtain the related S-parameters. The physical dimensions of the printed circuit board (PCB) were 30.75 mm long and 12.10 mm width. Figure 6 shows the layout of the PCB, with indications to the placement of the components. The parameters of a Rogers substrate (RO4003C, Rogers, Chandler, AZ, USA) were selected (*ε*_r_ = 3.55, tan*δ* = 0.0021, thickness = 1.524 mm).

**Figure 7:**
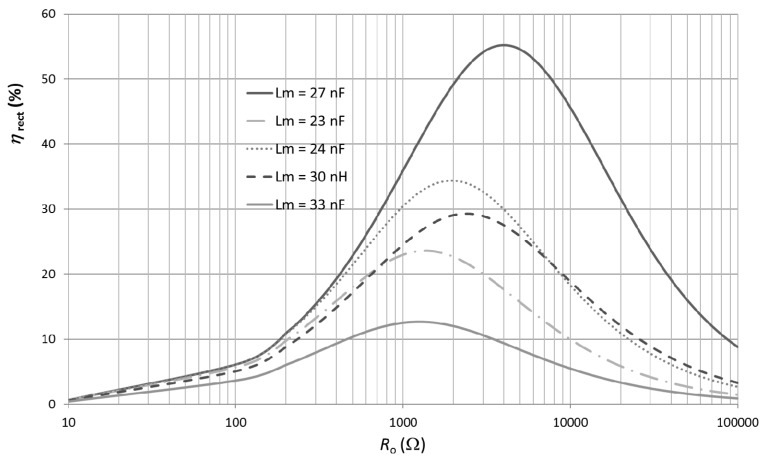
Simulation results of *η*_rect_ versus *R*_o_ for several values of *L*_m_ at *C*_m_ = 0.5 pF (*G*_t_ = 3.7) and *P*_av_ = −10 dBm.

**Figure 8:**
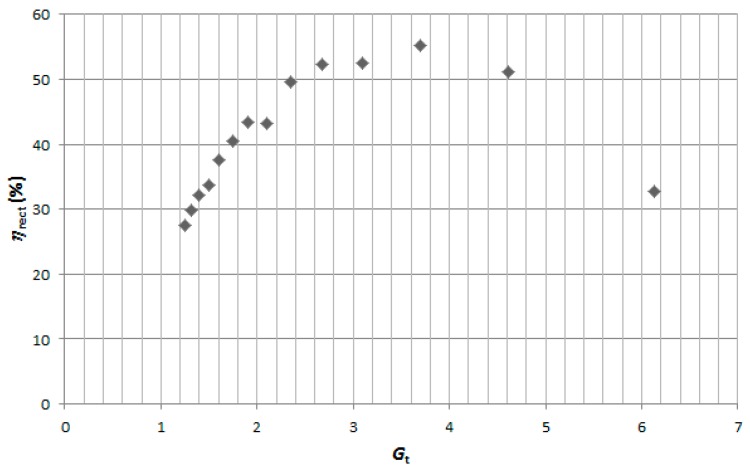
Simulation results of *η*_rect_ for several values of *G*_t_ at *P*_av_ = −10 dBm. A maximum value (*η*_max_) of 55.2% was achieved at *G*_t_ = 3.7 (*C*_m_ = 0.5 pF).

In order to find *G*_t,opt_ for each specific power level (*P*_av_), the following procedure was followed. First, an initial value of *G*_t_ is calculated using Equation (17) with appropriate values of *V*_γ_ and *R*_p_. From Equation (A5), the corresponding value of *C*_m_ is calculated and the component with the nearer commercial value is selected. Then, an appropriate component value of *L*_m_ is selected and a sweep of *η*_rect_ over *R*_o_ is performed. The procedure is repeated for several values of *L*_m_ until finding the curve with the maximum efficiency. In order to better illustrate the implemented procedure, Figure 7 shows the case for *P*_av_ = −10 dBm and *C*_m_ = 0.5 pF (*G*_t_ = 3.7). As can be seen, there is a maximum value of *η*_rect_ (around 55%) for *L*_m_ = 27 nH and *R*_o_ = 4 kΩ. These parameters values are saved and the whole procedure is repeated for different values of *C*_m_ (and thus of *G*_t_). Figure 8 shows the attained maximum efficiencies for *P*_av_ = −10 dBm for each one of the selected values of *C*_m_ (or *G*_t_). As can be seen, the maximum efficiency (*η*_max_) at −10 dBm was achieved for *G*_t_ = 3.7 *(C*_m_ = 0.5 pF, the case represented in Figure 7). Finally, the whole process is repeated for the different input power levels.

Table 1 summarizes the results of the simulations showing *η*_max_ along with the optimal values of *G*_t_, *C*_m_, *L*_m_, *R*_o_, and *V*_o_ for each one of the selected power levels (*P*_av_). As can be seen, the optimum value of *G*_t_ increases and *η*_max_ decreases with a decreasing value of *P*_av_ and thus of *V*_ap_, which agrees with the discussion of Section 2.2. As a numerical example, the value of *G*_t,opt_ for −20 dBm is calculated using Equation (17). Taking the data of the 27 nH inductor from Appendix B, it can be found from Equation (8) that *R*_p_ is 12.6 kΩ. Then, assuming a value of *V*_γ_ = 0.1 V, *G*_t,opt_ = 3.94 results (*C*_m_ = 0.47 pF). The value of *C*_m_ shown in Table 1 (0.5 pF) is in fact the nearest commercial value available from the vendor library.

## 4. Experimental Results and Discussion

**Figure 9:**
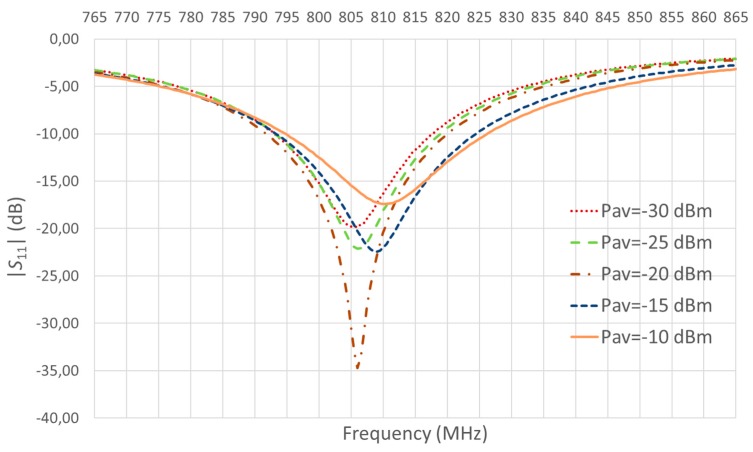
Experimental results of |*S*_11_|for different power levels.

The PCB layout of Figure 6 was produced and *C*_m_= 0.5 pF, *L*_m_ = 27 nH, and *C*_o_ = 1 nF were used. The selected values of *C*_m_ and *L*_m_ lead to *G*_t_ = 3.7 and match that of *P*_av_ = −20 dBm, −15 dBm and −10 dBm in Table 1. In order to choose an appropriate frequency for the experimental tests, the input reflection coefficient *S*_11_ of the rectenna was measured for *P*_av_ from −30 dBm to −10 dBm in steps of 5 dBm using for *R*_o_ the corresponding values of Table 1. Results are shown in Figure 9.

As can be seen, there is a deviation of the frequencies at which the minimum value is achieved with respect to the theoretical frequency of 868 MHz used in the simulations. This is probably due to differences between the models of the components used in the simulations and their actual values. The tolerance of the network components and deviations of the parasitic capacitances of the inductor and diode can be the main cause. In addition, the capacitance of the diode is nonlinear with the diode voltage drop and thus with *v*_in_, which accounts for the frequency shift down as the power decreases [8]. On the other hand, Table 2 shows the values of the input impedance of the rectenna at a frequency of 814 MHz, which was the value selected for the rest of tests. As can be seen, values approach the value of *R*_a_ = 50 Ω and, from Figure 9, |*S*_11_| was lower than −10 dB at that frequency for all power levels.

An RF signal generator was used at the input of the harvester to emulate the antenna and to generate different values of *P*_av_. Output power (*P*_o_) was measured by means of a Source Measurement Unit (SMU, B2901, Agilent, Santa Rosa, CA, USA). The frequency of the RF signal generator was set to 814 MHz, as previously stated, since it provided a relative high output power at all values of *P*_av_. Anyhow, other nearby frequencies (with a difference of few units of megahertz, e.g., 810 MHz or 805 MHz) could have been chosen without significant changes in *P*_o_.

While measuring *P*_o_, the SMU fixed the output voltage (*V*_o_) and this voltage was manually swept until the maximum value of *P*_o_ (*P*_o,max_) was obtained. Maximum efficiency (*η*_max_) was estimated as *P*_o,max_ divided by *P*_av_. Then, the equivalent value of *R*_o_ was estimated from *V*_o_ and *P*_o,max_. This procedure was faster than using a trimmer for *R*_o_ and estimating *P*_o_ from the measurements of *R*_o_ and *V*_o_ until *P*_o,max_ was obtained. Table 3 shows the values of *η*_max_, *R*_o_, and *V*_o_. Values are similar to that of the simulations (Table 1). Efficiencies range from 15.7% at −30 dBm to 55.2% at −10 dBm.

Table 4 shows a comparative of the rectenna efficiency (in percentage) of this work with other papers using similar designs. Some of the values are imprecise as they were inferred from graphs. All of them use a matching network (in most cases an L-type) and the same model of diode (except in [10] that use an HSMS-282X model (Avago Technologies). The frequency was similar (in the range of 850 MHz to 950 MHz) except in [10] (2.45 GHz), [20] (434 MHz), and [33] (1.8 GHz). As can be seen, this work outperforms the results of the rest of papers except at −30 dBm, where [20] presents a higher efficiency. It is possible that the losses of the inductor used here, which limit the network gain and efficiency, are higher than those of the inductor used in [20] (details of the commercial inductor not provided).

## 5. Conclusions

This work has demonstrated the existence of an optimum voltage gain for L-matching networks used in rectennas by providing an analytical expression. The rectenna, which also includes a Schottky single-diode rectifier, has been optimized at 868 MHz for a power range from −30 dBm to −10 dBm. As not all the parameters of the expression are well known a priori, an accurate search of the gain has been performed by simulations. Furthermore, a prototype has been implemented with experimental results showing remarkable power efficiencies, ranging from 16% at −30 dBm to 55% at −10 dBm. These results are amongst the highest published in the literature for similar designs.

## Figures and Tables

**Table 1:** Values of *η*_max_ along with the optimal values of *G*_t_, *C*_m_, *L*_m_, *R*_o_ and *V*_o_.

*P*_av_ (dBm)	*η*_max_ (%)	*G*_t_	*C*_m_ (pF)	*L*_m_ (nH)	*R*_o_ (kΩ)	*V*_o_ (mV)
−30	10.9	5.48	0.3	30	8.6	30.7
−25	18.6	5.48	0.3	30	7.0	64.2
−20	30.8	3.70	0.5	27	4.6	119
−15	44.6	3.70	0.5	27	4.4	249
−10	55.2	3.70	0.5	27	4.0	470

**Table 2:** Values of the input impedance of the rectenna at 814 MHz using for *R*_o_ the values of Table 1 .

*P*_av_ (dBm)	−30	−25	−20	−15	−10
Impedance (Ω)	67.3 + j22.4	65.9 + j18.8	59.6 + j10.7	60.7 − j9.9	62.3 − j11.7

**Table 3:** Experimental results of *η_max_*, *R*_o_, and *V*_o_.

*P*_av_ (dBm)	*η*_max_ (%)	*R*_o_ (kΩ)	*V*_o_ (mV)
−30	15.7	5.7	30
−25	24.6	4.6	60
−20	36.0	4.7	130
−15	47.2	4.5	260
−10	55.2	4.5	500

**Table 4:** Comparative of the rectenna efficiency (%) of this work with other papers with similar designs.

*P*_av_ (dBm)	This Work	[1]	[2]	[8]	[10]	[20]	[33]	[34]
−30	16	-	-	-	-	22	5	-
−25	25	-	-	20	-	-	8	-
−20	36	2	10	33	-	35	15	-
−15	47	5	20	42	-	-	25	30
−10	55	10	35	51	15	47	35	35

**Figure A1:**
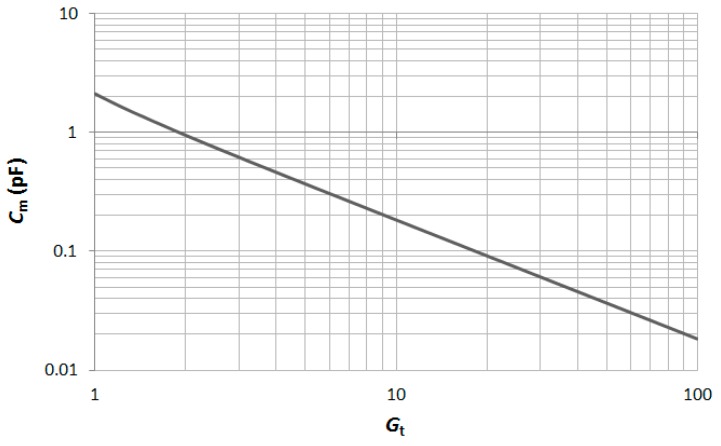
*C*_m_ values in function of *G*_t_.

**Figure A2:**
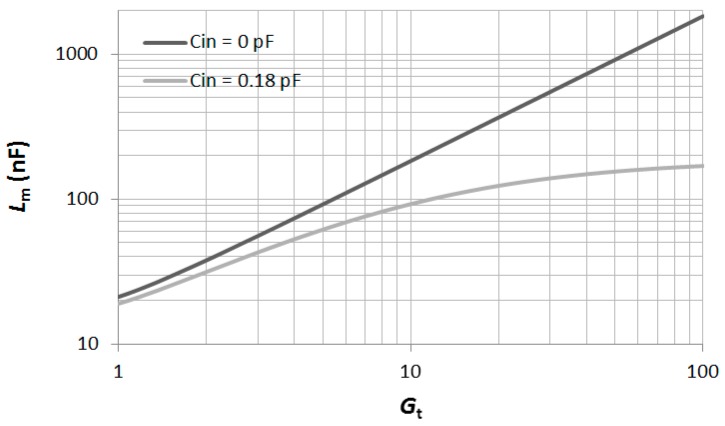
*L*_m_ values in function of *G*_t_ for *C*_in_ = 0 pF and *C*_in_ = 0.18 pF.

**Figure A3:**
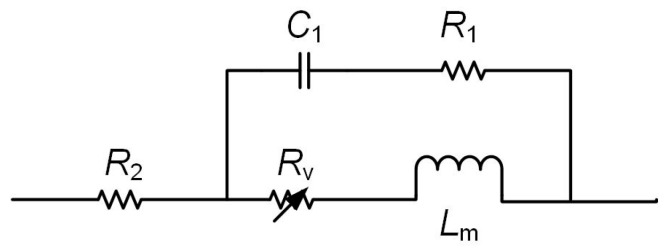
Equivalent lumped element model of the inductor.

**Figure A4:**
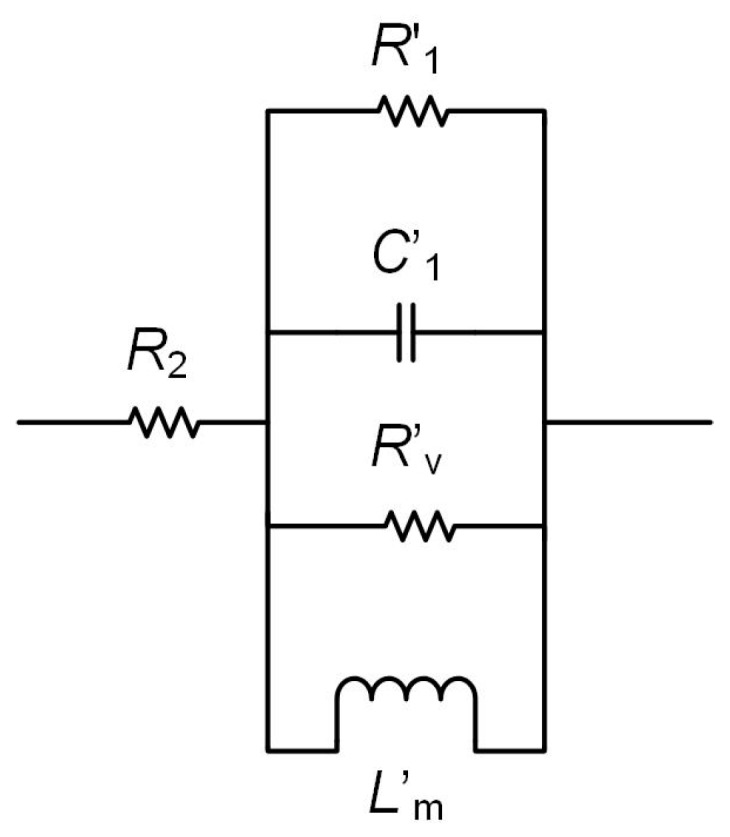
Parallel equivalent circuit of the inductor of Figure A3.

## Appendix A. Expressions for *C*_m_ and *L*_m_ and Corresponding Graphs

This appendix provides the expressions for *C*_m_ and *L*_m_ of the matching network along with graphs of these parameters.

In order to transfer the maximum power to the rectifier input, it must be accomplished that $Z_{L}=Z_{s}^{*}$ in Figure 3, where:

(A1) $$Z_{s}=R_{a}+\frac{1}{j\omega C_{m}},$$

(A2) $$\frac{1}{Z_{L}}=\frac{1}{R_{\mathrm{in}}}+j\omega C_{\mathrm{in}}+\frac{1}{j\omega L_{m}},$$

being *ω* the angular frequency. Operating, we obtain:

(A3) $$C_{m}=\frac{1}{\omega \sqrt{R_{a}\left(R_{\mathrm{in}}-R_{a}\right)}},$$

(A4) $$L_{m}=\frac{1}{\omega^{2}C_{\mathrm{in}}+\omega^{2}C_{m}\frac{R_{\mathrm{in}}-R_{a}}{R_{\mathrm{in}}}},$$

and the gain given by Equation (6). The parameters *L*_m_ and *C*_m_ can also be expressed in function of *G*_t_ and *Q* as:

(A5) $$C_{m}=\frac{1}{\omega R_{a}\;\sqrt{4\;G_{t}^{2}-1}}=\frac{1}{\omega R_{a}Q},$$

(A6) $$L_{m}=\frac{4\;G_{t}^{2}\;R_{a}}{\omega \;\sqrt{4\;G_{t}^{2}-1}+4\;G_{t}^{2}\;R_{a\;}C_{in}\;\omega^{2}}=\frac{1}{\omega^{2}}\frac{1}{C_{\mathrm{in}}+C_{m}Q^{2}/\left(1+Q^{2}\right)}.$$

Figure A1 and Figure A2 show the values of *C*_m_ and *L*_m_ as a function of *G*_t_ for *R*_a_ = 50 Ω, respectively. As for the calculus of *L*_m_, two different cases have been considered, *C*_in_ equal to 0 pF and to 0.18 pF. The value of 0.18 pF emulates the input capacitance of the selected diode (HSMS-2850) for the rectenna (Section 3 and Section 4). *C*_m_ is inversely proportional to *G*_t_ (strictly only for *G*_t_ >> 1) and *Q*. On the other hand, *L*_m_ is inversely proportional to *C*_m_ (so, proportional to *G*_t_ and *Q*) for *C*_in_ = 0 but for *C*_in_ = 0.18 pF saturates to $1/\left(\omega^{2}C_{\mathrm{in}}\right)$ with *G*_t_ (or *Q*) increasing (*C*_m_ decreasing).

## Appendix B. Lumped Model of the Inductor Including Parasitic Effects

Figure A3 shows the lumped model of the Coilcraft RF surface mount inductor used in the implementation of the circuit of Figure 2. The value of the frequency-dependent variable resistor *R*_v_ relates to the skin effect and is calculated from $R_{v}=k^{\prime }\sqrt{f}$, where *f* is the operation frequency and *k*’ is a constant that depends on the nominal value of the inductance.

Using the series to parallel equivalent circuit transformation for the two branches of Figure A3 the equivalent circuit of Figure A4 results.

The value of the components of the circuit of Figure A4 respect to those of the circuit of Figure A3 are given by the following expressions:

(A7) $${R^{\prime }}_{1}=R_{1}\frac{R_{1}^{2}C_{1}^{2}\omega^{2}+1}{R_{1}^{2}C_{1}^{2}\omega^{2}},$$

(A8) $${C^{\prime }}_{1}=\frac{C_{1}}{R_{1}^{2}C_{1}^{2}\omega^{2}+1},$$

(A9) $${L^{\prime }}_{m}=L_{m}\left(1+{\left(\frac{R_{v}}{\omega L_{m}}\right)}^{2}\right),$$

(A10) $${R^{\prime }}_{v}=R_{v}\left(1+{\left(\frac{\omega L}{R_{v}}\right)}^{2}\right).$$

The model of Figure A4 is substituted in the circuit of Figure 3, resulting in the circuit of Figure 4, where the value of *R*_2_ has been neglected as it usually is very small. As an example, the 27 nH inductor used in the implemented matching network (Section 4) has the following parameters: *R*_1_ = 17 Ω, *R*_2_ = 30 mΩ, *C*_1_ = 49 fF, *L*_m_ = 27 nH, *k*’ = 5.75 × 10^−5^. At 868 MHz the value of the parallel components of Figure A4 are: $R_{1}^{\prime }=824\;k\Omega$, $C_{1}^{\prime }=49\;\mathrm{fF}$, $R_{v}^{\prime }=12.8\;k\Omega$, $L_{m}^{\prime }=27\;\mathrm{nH}$.
